# Chronic traumatic encephalopathy pathognomonic lesions occurring in isolation adjacent to infiltrative and non-infiltrative white matter lesions

**DOI:** 10.1093/jnen/nlae046

**Published:** 2024-05-15

**Authors:** Michaela M Scanlon, Margaret M Shields, Daniel P Perl, David S Priemer

**Affiliations:** F. Edward Hébert School of Medicine, Uniformed Services University, Bethesda, Maryland, USA; F. Edward Hébert School of Medicine, Uniformed Services University, Bethesda, Maryland, USA; F. Edward Hébert School of Medicine, Uniformed Services University, Bethesda, Maryland, USA; Department of Defense/Uniformed Services University Brain Tissue Repository, Uniformed Services University, Bethesda, Maryland, USA; F. Edward Hébert School of Medicine, Uniformed Services University, Bethesda, Maryland, USA; Department of Defense/Uniformed Services University Brain Tissue Repository, Uniformed Services University, Bethesda, Maryland, USA

**Keywords:** Age-related tau astrogliopathy, Chronic traumatic encephalopathy, Tau, Traumatic brain injury, White matter

## Abstract

Chronic traumatic encephalopathy (CTE) is defined by perivascular neuronal phosphorylated-tau accumulation at cortical sulcal depths. CTE has been mainly described in the context of repetitive, impact-type traumatic brain injury (rTBI), principally from contact sports. Rarely, CTE has been associated with single TBIs, including in relationship to healed leucotomy sites in brains from formerly institutionalized psychiatric patients without documented rTBI. Given that leucotomy principally involves severing of white matter, this could suggest involvement of axonal injury in CTE pathophysiology. We present three cases wherein isolated CTE pathology was identified adjacent to distinct white matter lesions. Case 1 is a 41-year-old man with history of hereditary hemorrhagic telangiectasia and resection of a cerebral arteriovenous malformation (AVM). Case 2 is a 46-year-old man with glioblastoma. Case 3 is a 52-year-old man with a remote cerebral infarct. Isolated CTE lesions were found adjacent to the aforementioned pathologies in each case. Additional CTE lesions were not identified despite extensive sampling. Multiple age-related tau astrogliopathy (ARTAG)-like lesions were also identified at other sulcal depths near the AVM resection site in Case 1. These cases may provide insights regarding the pathophysiology of the CTE pathognomonic lesion and the development of ARTAG-like pathology adjacent to long-standing mass lesions.

## INTRODUCTION

Chronic traumatic encephalopathy (CTE) is a secondary tauopathy most commonly associated with repetitive traumatic brain injury (rTBI). Currently, CTE can only be diagnosed by postmortem brain examination through the identification of a “CTE pathognomonic lesion,” i.e. phosphorylated tau aggregates in neurons, with or without thorn-shaped astrocytes, at the depth of a cortical sulcus in a perivascular distribution and in deep cortical layers (1). A CTE lesion should not demonstrate tau aggregates that are restricted to the subpial and superficial region of the sulcus. CTE is most often described in the setting of long-standing participation in contact sports, principally American football and boxing, though it has been associated with other sports including soccer, hockey, and even baseball (2–4). CTE has also been described in other settings of rTBI such as domestic violence, epileptic seizures, and in patients with neurodevelopmental disorders who exhibited head-banging behaviors (5, 6). However, CTE-like pathology has also been associated with the context of single, severe TBIs, to include occurring adjacent to healed leucotomy (“lobotomy”) sites in the brains of institutionalized psychiatric patients who had no documented history of rTBI (7, 8). Interestingly, the leucotomy procedure, which is a singular iatrogenic TBI performed bilaterally in the prefrontal cortex, principally involves destruction of subcortical white matter as opposed to cerebral cortex, and does not involve any sort of impact head injury. The finding of CTE pathology adjacent to focal white matter lesions may have important implications with regard to potential mechanisms that may underlie CTE lesion development.

Herein, we present three autopsy cases in which a CTE pathognomonic lesion was identified in the cerebral cortex immediately adjacent to underlying infiltrative and non-infiltrative white matter pathology. These cases were received as donations to the Department of Defense/Uniformed Services University Brain Tissue Repository.

## CASE HISTORIES

### Case 1

The decedent was a 41-year-old man with a history of hypertension, coronary artery disease, myocardial infarction, deep venous thrombosis, obstructive sleep apnea, and prior alcohol abuse. He also had a diagnosis of hereditary hemorrhagic telangiectasia (Osler-Weber-Rendu Syndrome), and was approximately 6 years status post surgical resection of an arteriovenous malformation (AVM) that had presented with intracerebral hemorrhage and was located in the right frontotemporal region. Of note, he was a former Service Member, having served in the Navy for 5 years, but without reported combat deployment or injuries. He played baseball and soccer during his school years. Approximately one year prior to death, he was involved in a motor vehicle accident and sustained a TBI associated with loss of consciousness. He died of an acute pulmonary embolism secondary to deep venous thrombosis.

### Case 2

The decedent was a 46-year-old man with a history of glioblastoma that was diagnosed less than 1.5 years prior to death. He was status post craniotomy for resection and chemoradiation therapy. His clinical course was complicated by seizures, tremors, and progressive unilateral weakness. He died of complications from the malignancy. Of note, he was an active duty Service Member in the Special Forces with a history of numerous combat deployments. It was reported to us that he sustained service-related TBIs but further details are unknown. The decedent had a history of participation in boxing, the extent of which is also unknown.

### Case 3

The decedent was a 52-year-old man with a history of type II diabetes mellitus, peripheral neuropathy, hyperlipidemia, hypertension, coronary artery disease, obstructive sleep apnea, and remote cerebral infarction that resulted in left-sided paraplegia. Of note, he was a military veteran, having served in the Gulf War and in Operation Desert Storm as a heavy machine operator. He was diagnosed with post-traumatic stress disorder and depression. He was also a former athlete, having wrestled in high school and played American Football in high school, college (1 year), and recreationally while in the military. He died of an acute myocardial infarction.

## NEUROPATHOLOGICAL EXAMINATION

Whole, formalin-fixed, brains were evaluated for all of the described cases. Complete gross examination and sampling included bilateral cerebral hemispheres, the brainstem, and the cerebellum. In all cases, sampling for microscopic examination included of the orbitofrontal region (bilateral), dorsolateral prefrontal region (bilateral), the superior and middle temporal gyri, the inferior parietal lobule (bilateral), the visual cortex, the anterior insula, the cingulate gyrus, the amygdala and adjacent mesial temporal cortex, the hippocampi with adjacent mesial temporal cortex (bilateral), basal ganglia (bilateral), midbrain, pons, and cerebellum with dentate nucleus. Additional samples related to the AVM resection cavity were taken in Case 1 (five additional in total), related to the gross malignancy in Case 2 (six additional in total), and related to the remote cerebral infarct in Case 3 (three additional in total), such that were a total of 19 cerebral cortex-containing samples in Case 1, 20 in Case 2, and 17 in Case 3. It should be noted that this extent of sampling exceeds current consensus recommendations regarding cortical sampling for initial screening for CTE and also secondary sampling in suspicious cases (1, 9).

**Figure 1.**
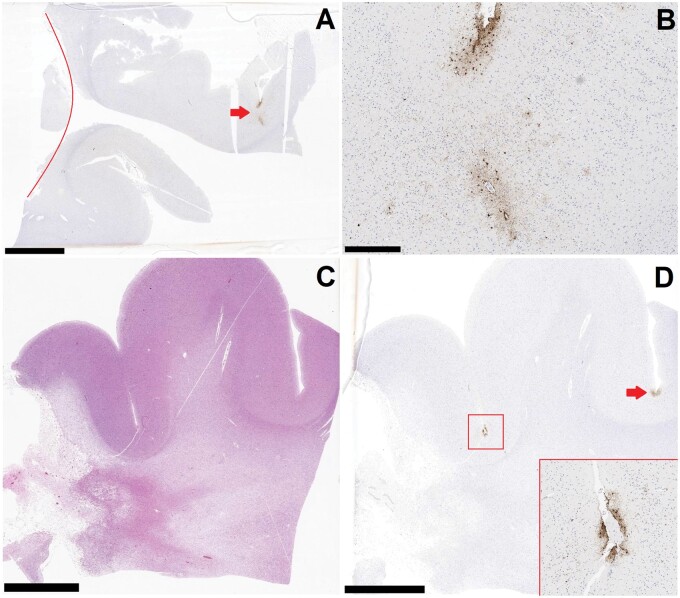
Chronic traumatic encephalopathy (CTE) and age-related tau astrogliopathy (ARTAG)-like pathology adjacent to the resection cavity of a prior arteriovenous malformation. **(A)** Low-magnification (scale bar = 5 mm) image of temporal lobe sample, with resection cavity approximated by the red line. AT8 immunohistochemical stain identifies a pathognomonic lesion for CTE at an adjacent sulcal depth (red arrow). **(B)** High magnification (scale bar = 500 µm) of the lesion in (**A**), AT8 immunostain. **(C)** Low-magnification (scale bar = 5 mm) H&E photomicrograph demonstrating the gliotic AVM resection site in the white matter. **(D)** Low-magnification (scale bar = 5 mm) of the AT8 immunohistochemical stain corresponding to **(C)**, demonstrating several sulcal-depth lesions most characteristic of ARTAG. The higher magnification inset corresponds to boxed the ARTAG lesion. The red arrow denotes another ARTAG lesion in the neighboring sulcus.

**Figure 2.**
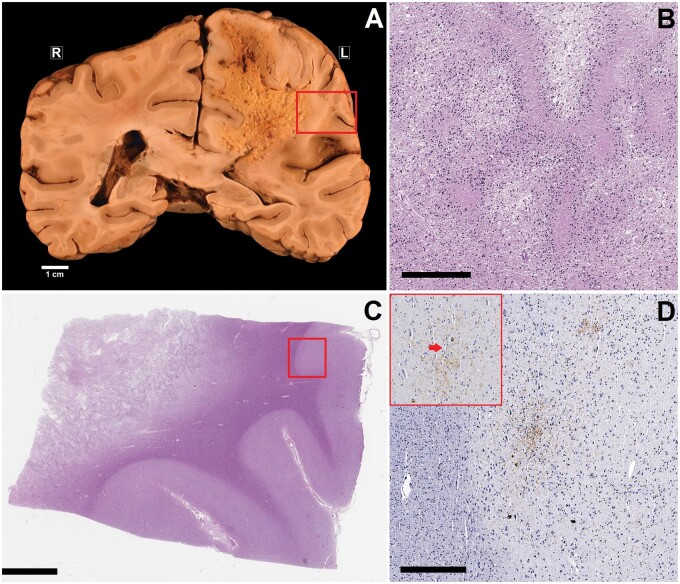
Chronic traumatic encephalopathy (CTE) pathology adjacent to a glioblastoma. **(A)** Gross photograph demonstrating infiltrative malignancy in the left cerebral hemisphere (parietal region), with some visible extension into the corpus callosum. The red box indicates where a sample was taken for histology, the sections from which are shown in (**B–D**). **(B)** Representative photomicrograph of the glioblastoma, demonstrating characteristic serpentine necrosis with nuclear pseudopalisading (scale bar = 300 µm). **(C)** Low-magnification (scale bar = 4 mm) H&E photomicrograph demonstrating the cerebral tissue sample with infiltrative tumor in the subcortical white matter. The red box indicates the area of focus for (**D**). **(D)** AT8 immunohistochemical stain corresponding to the region highlighted by the red box in (**C**), demonstrating a sulcal depth lesion with neuronal tau surrounding a small blood vessel (scale bar = 400 µm). The high magnification inset demonstrates the small vessel (arrow).

**Figure 3.**
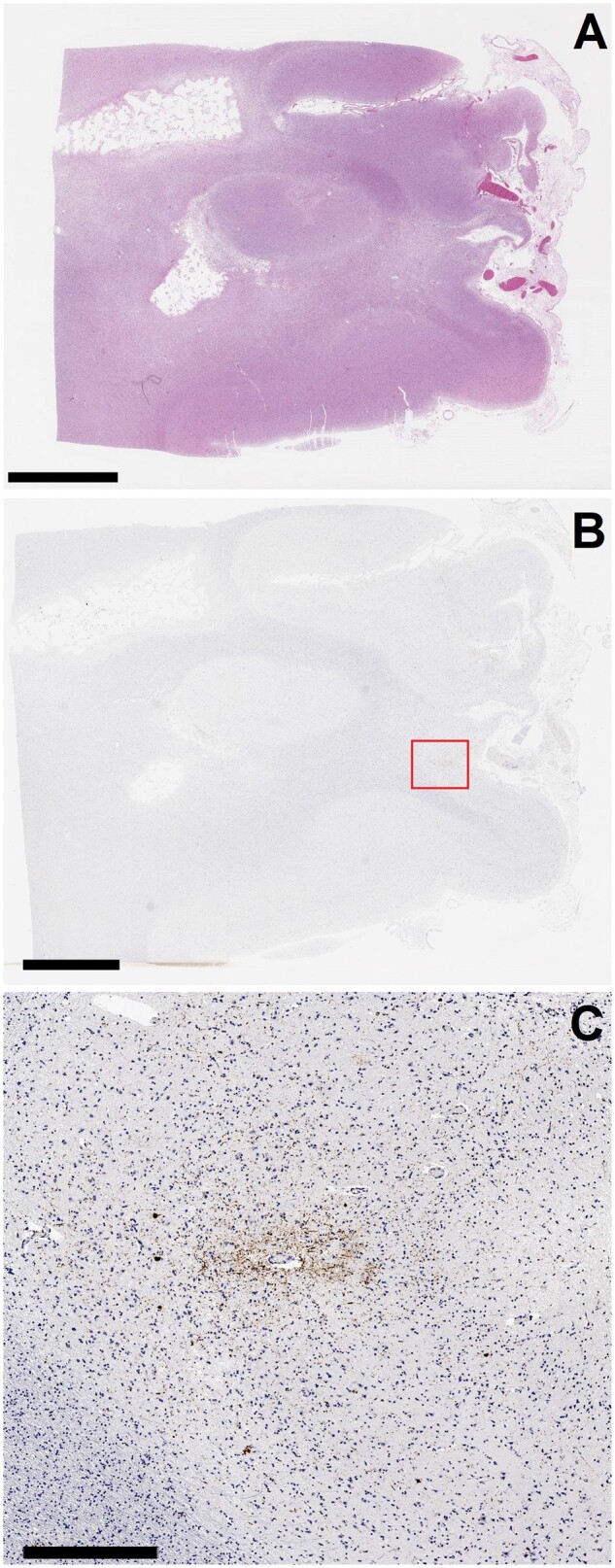
Chronic traumatic encephalopathy (CTE) pathology adjacent to a remote infarct primarily involving white matter. **(A)** Low magnification (scale bar = 5 mm), H&E photomicrograph of remote, healed infarct with large areas of white matter cavitation and gliosis, and focal cortical loss. **(B)** Low magnification (scale bar = 4 mm) photomicrograph of the AT8 immunohistochemical stain corresponding to a section from the same block as from (**A**). The red box indicates the region of interest for (**C**). **(C)** AT8 immunohistochemical stain corresponding to the region highlighted by the red box in **(B)**, showing a collection of neuronal tau around a small vessel in the sulcal depth (scale bar = 500 µm).

Gross and hematoxylin and eosin (H&E) microscopic examination of all 3 cases was compatible with the clinical histories/diagnoses. A gliotic resection cavity involving the white matter in the right frontotemporal region was identified in Case 1, without any residual evidence of an AVM (Fig. 1A, C). A large, high-grade glioma with histology compatible with glioblastoma, occupying much of the left cerebral hemisphere and with extension across the corpus callosum into the right cerebral hemisphere, was identified in Case 2 (Fig. 2A, B). Multiple areas of cavitated, remote cerebral infarction were identified in the brain of Case 3, in the right cerebral hemisphere and in a cerebral artery border zone distribution; in particular, an area of remote infarction in the right dorsolateral prefrontal region involved sizable gliotic cavitation in the white matter and only focally involved the overlying cerebral cortex (Fig. 3A).

Immunohistochemistry for phosphorylated tau (AT8 mouse anti-human monoclonal antibody [MN1020]; Thermo Scientific, Waltham, MA) was performed on sections from all sampled regions. In the tau-stained sections, A total of 51 cortical sulcal depths were ultimately evaluated in Case 1, 50 in Case 2, and 55 in Case 3. Examination in all 3 cases revealed an isolated CTE pathognomonic lesion immediately adjacent to the underlying white matter pathology. Additional, or more distant CTE lesions were not identified in any of the 3 cases. In Case 1, beyond the CTE lesion (Fig. 1A, B), other areas of cortex sampled from near the resection cavity in the right frontotemporal region demonstrated several astrocytic tau accumulations in the subpial/superficial region of the cortex, histologically compatible with a diagnosis of age-related tau astrogliopathy (ARTAG) in sulcal depths (Fig. 1D). Occasional neuronal tau aggregation (no sulcal depth predominance) was also identified in the cortex adjacent to the resection cavity. Cortical neuronal tau aggregation was not identified in neocortex sampled away from the AVM resection cavity. The right amygdala, hippocampus, and entorhinal region demonstrated minimal neurofibrillary changes; there were a few tau-positive neurons and/or neuronal threads that were not observed in sections from the left side. In Case 2, the CTE lesion was identified overlying white matter infiltration by glioblastoma in the left parietal lobe (Fig. 2C, D). Apart from the isolated CTE lesion in Case 2, there was minimal neurofibrillary pathology in the entorhinal regions and hippocampus bilaterally; other cortical tau included a small focus of mid-cortical glial and neuronal tau aggregation within cortex infiltrated by malignancy. In Case 3, apart from an isolated CTE lesion identified adjacent to the aforementioned infarction in the right dorsolateral prefrontal region (Fig. 3B, C), cortical tau pathology included rare and isolated neuronal tau aggregates identified in samples of the frontal and temporal neocortices, the parietal cortices, the entorhinal regions, and the hippocampi.

Immunohistochemistry for Aβ protein (4G8, mouse anti-human monoclonal antibody; Covance/BioLegend, Princeton, NJ) was also performed on sections from all sampled regions. There were occasional single or small groups of diffuse-type Aβ deposits in the cortex of all 3 cases (Thal phase 1), without a predilection for the areas of underlying white matter pathology. Neuritic plaques were not identified in any of the 3 cases (CERAD score 0). Immunohistochemistry for α-synuclein (recombinant anti-alpha synuclein [Phosphor S129] antibody [EP1536Y]; Abcam, Waltham, MA) and TDP-43 (recombinant anti-TDP 43 antibody [EPR 5810]; Abcam) was performed on sections from a subset of locations (α-synuclein: cingulate gyrus, dorsolateral prefrontal cortex, superior and middle temporal gyri, amygdala and adjacent mesial temporal cortex, midbrain, and pons; TDP-43: amygdala with adjacent mesial temporal cortex, hippocampus with adjacent mesial temporal cortex, super and middle temporal gyri, dorsolateral prefrontal cortex). These immunohistochemical stains were negative in all 3 cases. Additional photomicrographs from the 3 cases are in Supplementary Data Figs 1–3.

## DISCUSSION

Given the function of tau as a microtubule-associated protein that is involved in the stability of axonal microtubules in white matter, it has been suggested that axonal injury may predispose to the characteristic tau accumulation pattern of CTE (8, 10, 11). Though a controversial issue, this would seem to be supported in the literature by the rare association of CTE with single, severe TBIs, including with leucotomy sites in psychiatric patients who had been institutionalized (8). In this series we describe three cases in which the cerebral cortex was extensively sampled and revealed an isolated pathognomonic lesion for CTE. In each of these three cases, the CTE lesion was identified adjacent to an infiltrative or non-infiltrative lesion involving the white matter. CTE lesions elsewhere were not identified. Cortical tau pathology apart from the isolated CTE lesions in each case was minimal and is likely in most part to reflect early age-related changes (12–15). While neurofibrillary changes adjacent to arteriovenous malformations and other vascular pathologies have been previously reported (16–18), these have not been described with the specific features of CTE. Some neurofibrillary changes were identified in the right mesial temporal lobe in Case 1, which was the same lobe involved by the prior AVM, while these changes were not identified in samples from the left side. It is difficult to speculate whether these neurofibrillary changes correspond to the AVM or its resection, or if they represent asymmetric age-related pathology (or some combination of these factors) (19). A review of the peer-reviewed literature does not does not reveal a previously-described association of CTE pathology in the setting of an underlying diffuse glioma or cerebral infarct. It is important to note that all three of the cases we present involved some degree of prior contact sport exposure, and all involved former Military Service Members; thus, a relationship with these histories and CTE cannot be excluded. However, the location of the CTE lesions in these cases as isolated to overlying an area of white matter pathology and not identified away from those lesions is of particular interest. It is possible that there is a compounding effect between prior TBI and these isolated lesions, i.e. such white matter injury may potentiate CTE development from rTBI, or vice versa. As such, these cases may provide further insight regarding the nature of the CTE pathognomonic lesion and its potential relationship to white matter injury.

The current consensus recommendations regarding a sampling protocol for the primary assessment of CTE pathology include eleven sites, five of which are cerebral cortex-containing and therefore could show diagnostic CTE as currently defined: middle frontal gyrus, superior and middle temporal gyri, inferior parietal lobule, hippocampus, and amygdala (1, 9). When CTE is suspected, presumably from either patient history or the initial set of samples, but a pathognomonic lesion is not identified, some degree of further sampling is recommended, to include superior frontal, dorsolateral superior frontal, superior middle temporal, and/or inferior temporal regions. The extent of cortical sampling in our attempts to identify additional CTE lesions in the three cases presented far exceeded these recommended protocols, with 17 or more cortex-containing samples having been examined in each case. While it remains possible that further sampling would reveal additional CTE pathology, there is currently no guidance regarding the upper limitations of such a pursuit. There are also no published guidelines regarding a minimum threshold of cortical sulcal depths needed to comprehensively evaluate a case of CTE, however in the three cases presented here there were 50 or more cortical sulcal depths examined.

Another finding within the series was that of numerous, superficially oriented astrocytic tau lesions involving multiple sulcal depths adjacent to the AVM resection cavity site in Case 1. These lesions were separate from the identified CTE lesion, and do not meet criteria as pathognomonic lesions for CTE, but rather are most diagnostic of ARTAG. The relationship of CTE to astrocytic tau pathology has been a matter of significant debate, particularly since the first CTE neuropathology consensus diagnostic criteria were published in 2016 (20). Although the presence of astrocytic tau has been de-emphasized in the current diagnostic criteria for CTE, researchers have demonstrated that sulcal depth astrocytic tau deposition is nonetheless common in cases of CTE, and helps in its recognition as a diagnostic entity in clinical practice (21). Nonetheless, recent research conducted by Butler et al specifically on the relationship of CTE and sulcal depth ARTAG, and of them both to a history of rTBI, suggested that the presence of ARTAG is most strongly a function of age rather than TBI history (22). Further, the pathology of ARTAG, as can be inferred by the name, is generally regarded as a pathology seen in aged individuals. Animal studies have also not shown an association between TBI and the development of ARTAG-like pathology (23). However, of interest to this recent research, Case 1 is that of a 41-year-old individual who has ARTAG-like pathology that was found in the immediate vicinity of the resection site of an AVM. This finding corroborates suggestions made in the literature that chronic, space-occupying lesions in the brain can trigger astrocytic tau aggregation befitting of ARTAG as a result of longstanding mechanical stress (24), and demonstrates that this pathological finding can be seen in young-to-middle ages.

In conclusion, we present three cases in which there is an isolated CTE pathognomonic lesion immediate adjacent to a distinct white matter pathology, and wherein extensive cortical sampling did not reveal additional CTE sulcal depth lesions. While recognizing that the cases involve individuals with some exposure to rTBI, the location of the CTE lesions as limited to overlying a distinct white matter pathology may suggest involvement of white matter injury in the pathobiology of the characteristic tau pathology of CTE. Further studies examining additional cases with similar white matter disruptions are warranted.

## FUNDING

Funding support for this article was provided by the Department of Defense/Uniformed Services University Brain Tissue Repository and Neuropathology Program (HU00012120007).

## DISCLAIMER

The information, content, conclusions, and/or opinions expressed herein do not necessarily represent the official position or policy of, nor should any official endorsement be inferred on the part of, Uniformed Services University, the Department of Defense, or the United States Government.

## Supplementary Material

nlae046_Supplementary_Data
