# Supplementary material for: Chronic traumatic encephalopathy pathognomonic lesions occurring in isolation adjacent to infiltrative and non-infiltrative white matter lesions
Source: J Neuropathol Exp Neurol. 2024 May 15;83(8):695–700. doi: 10.1093/jnen/nlae046 (PMC11258416; doi:10.1093/jnen/nlae046)
Supplement: nlae046_Supplementary_Data [file nlae046_supplementary_data.zip › nlae046_Supplementary_Data/Rays redone Priemer Supplementary Figure 1 with Legend.PPTX]

## Slide 1
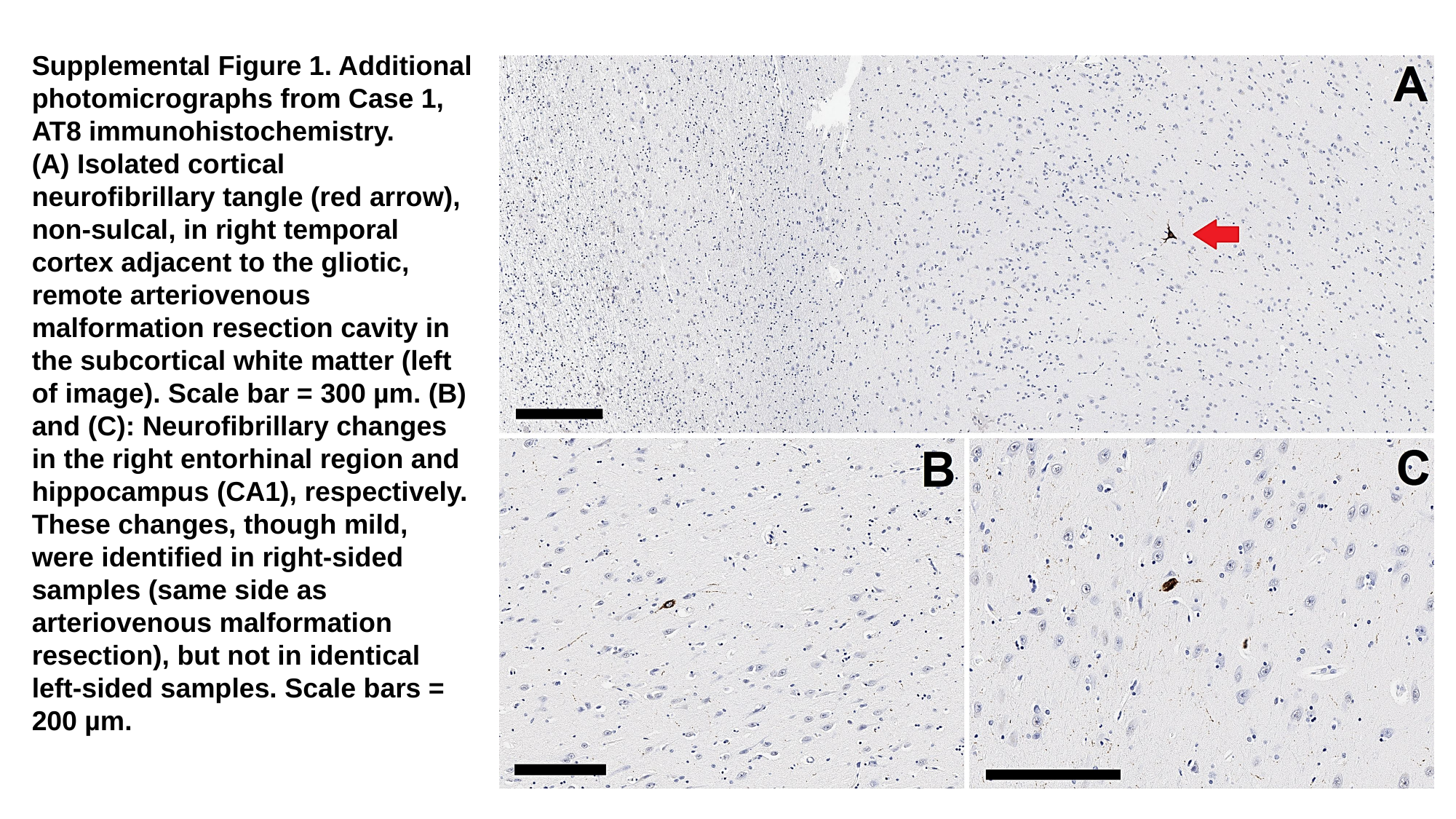

Supplemental Figure 1. Additional photomicrographs from Case 1, AT8 immunohistochemistry.
(A) Isolated cortical neurofibrillary tangle (red arrow), non-sulcal, in right temporal cortex adjacent to the gliotic, remote arteriovenous malformation resection cavity in the subcortical white matter (left of image). Scale bar = 300 µm. (B) and (C): Neurofibrillary changes in the right entorhinal region and hippocampus (CA1), respectively. These changes, though mild, were identified in right-sided samples (same side as arteriovenous malformation resection), but not in identical left-sided samples. Scale bars = 200 µm.
