# Supplementary material for: Chronic traumatic encephalopathy pathognomonic lesions occurring in isolation adjacent to infiltrative and non-infiltrative white matter lesions
Source: J Neuropathol Exp Neurol. 2024 May 15;83(8):695–700. doi: 10.1093/jnen/nlae046 (PMC11258416; doi:10.1093/jnen/nlae046)
Supplement: nlae046_Supplementary_Data [file nlae046_supplementary_data.zip › nlae046_Supplementary_Data/Rays redone Priemer Supplementary Figure 2 with Legend.PPTX]

## Slide 1
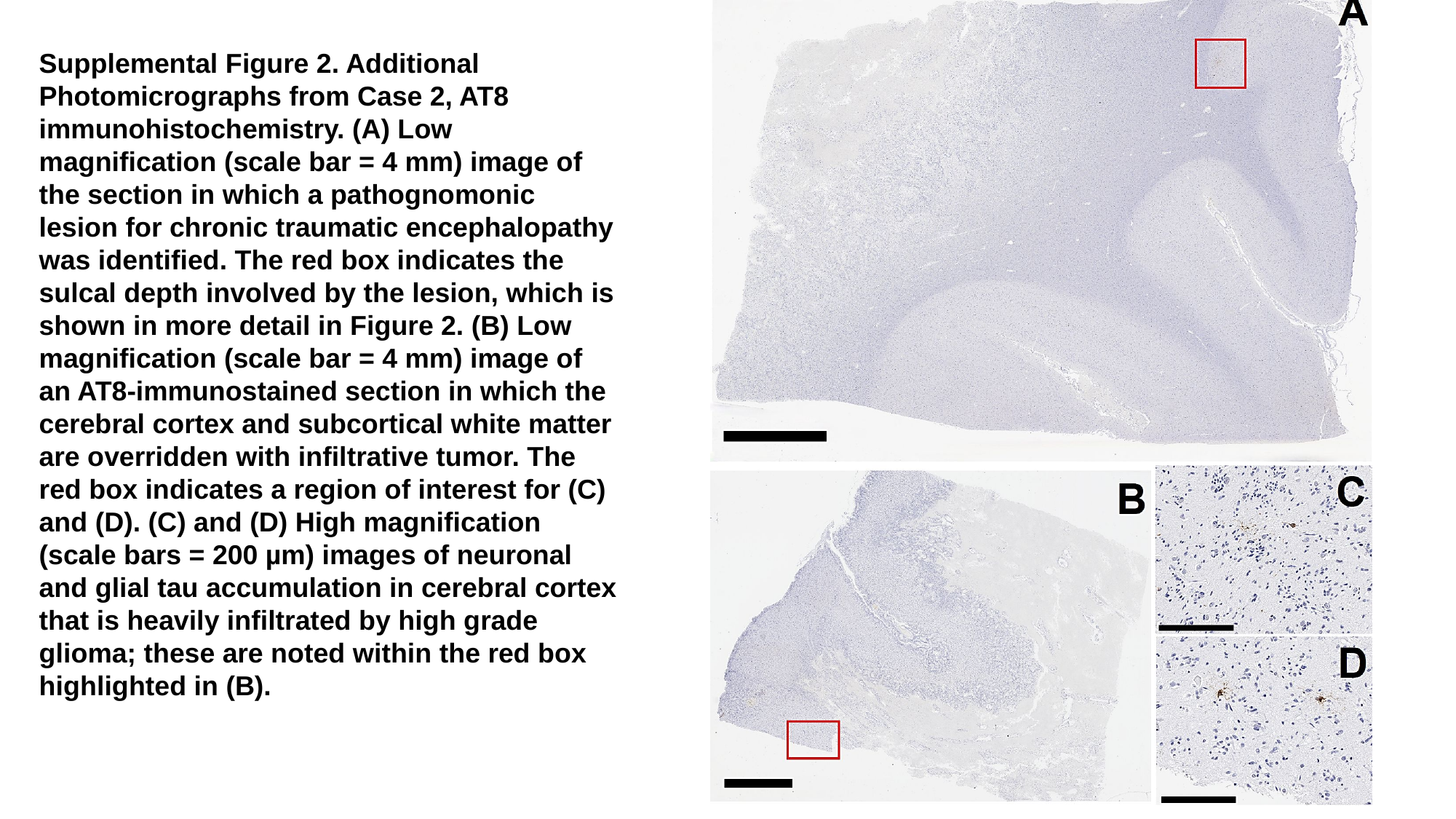

Supplemental Figure 2. Additional Photomicrographs from Case 2, AT8 immunohistochemistry. (A) Low magnification (scale bar = 4 mm) image of the section in which a pathognomonic lesion for chronic traumatic encephalopathy was identified. The red box indicates the sulcal depth involved by the lesion, which is shown in more detail in Figure 2. (B) Low magnification (scale bar = 4 mm) image of an AT8-immunostained section in which the cerebral cortex and subcortical white matter are overridden with infiltrative tumor. The red box indicates a region of interest for (C) and (D). (C) and (D) High magnification (scale bars = 200 µm) images of neuronal and glial tau accumulation in cerebral cortex that is heavily infiltrated by high grade glioma; these are noted within the red box highlighted in (B).
