# Supplementary material for: Chronic traumatic encephalopathy pathognomonic lesions occurring in isolation adjacent to infiltrative and non-infiltrative white matter lesions
Source: J Neuropathol Exp Neurol. 2024 May 15;83(8):695–700. doi: 10.1093/jnen/nlae046 (PMC11258416; doi:10.1093/jnen/nlae046)
Supplement: nlae046_Supplementary_Data [file nlae046_supplementary_data.zip › nlae046_Supplementary_Data/Ray[s redone Priemer Supplementary Figure 3 with Legend.PPTX]

## Slide 1
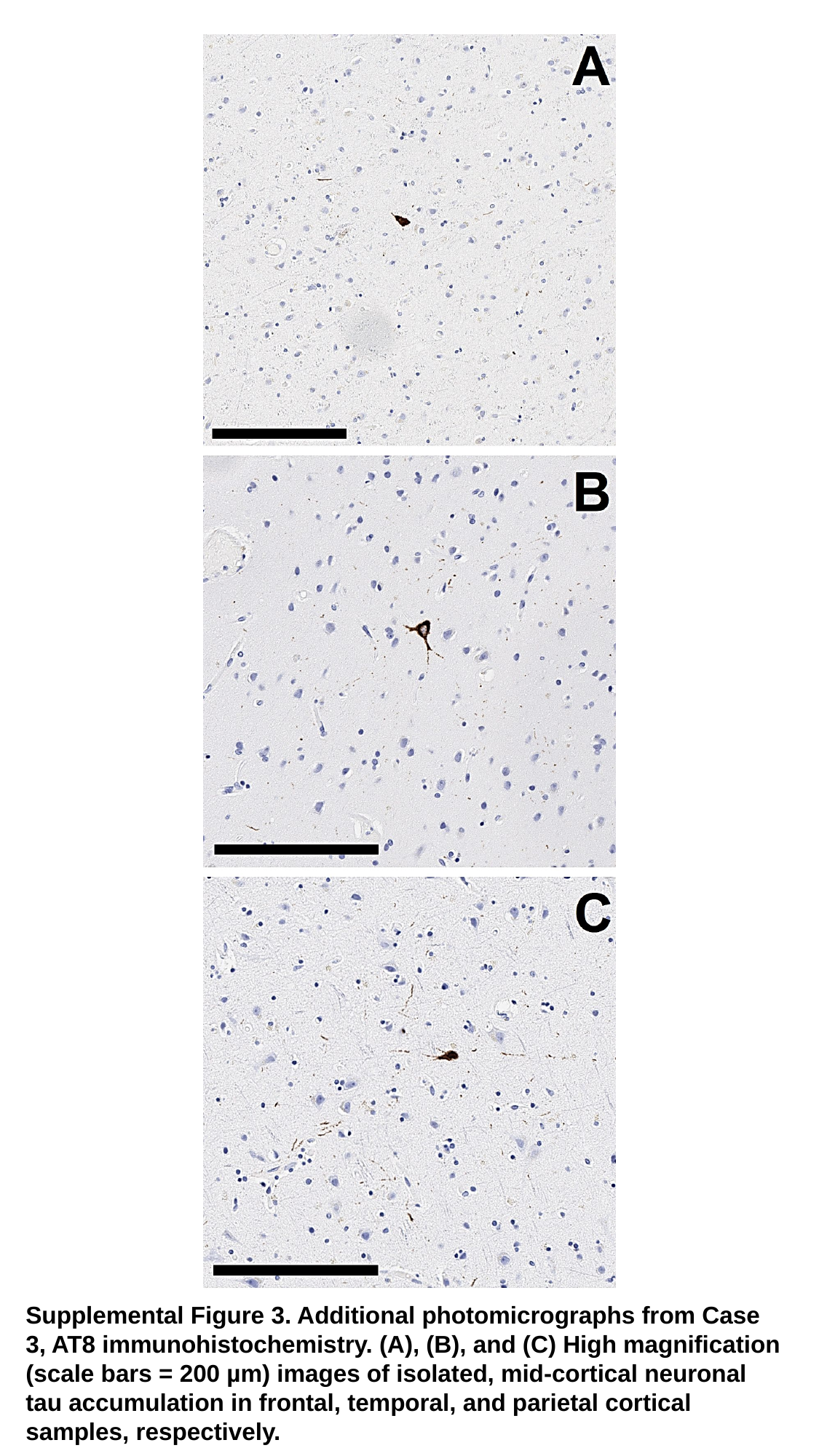

Supplemental Figure 3. Additional photomicrographs from Case 3, AT8 immunohistochemistry. (A), (B), and (C) High magnification (scale bars = 200 µm) images of isolated, mid-cortical neuronal tau accumulation in frontal, temporal, and parietal cortical samples, respectively.
